# Curious Case of a Giant Retroperitoneal Cyst

**DOI:** 10.7759/cureus.51758

**Published:** 2024-01-06

**Authors:** Viral Surani, Sanjay Chatterjee, Sunila Jaggi, Niranjan Banka, Shivani Desai, Gajanan Rodge

**Affiliations:** 1 Gastroenterology and Hepatology, Bombay Hospital and Medical Research Center, Mumbai, IND; 2 Surgery, Bombay Hospital and Medical Research Center, Mumbai, IND; 3 Radiology, Bombay Hospital and Medical Research Center, Mumbai, IND; 4 General Surgery, Bombay Hospital Institute of Medical Sciences, Mumbai, IND

**Keywords:** uniloculated cyst, giant cyst, serous fluid, adrenal cyst, retroperitoneal cyst

## Abstract

Adrenal cysts are uncommon fluid-filled masses that develop in the adrenal gland. Typically, they are non-functional, asymptomatic, and smaller than 10 cm in diameter when incidentally detected. However, the presence of giant adrenal cysts, exceeding 10 cm in diameter, creates a diagnostic challenge due to the difficulty in determining their origin. Surgical intervention is advised when the cyst surpasses 10 cm in diameter, produces symptoms, causes endocrine abnormalities, exhibits intracystic bleeding, or raises suspicion of malignancy. The preferred treatment approach involves adrenalectomy, performed either through open surgery or laparoscopy. In cases where the diagnosis is unequivocal, ultrasound-guided percutaneous drainage serves as an alternative. Here, we present an exceptional case of a massive retroperitoneal mass caused by a rare giant adrenal cyst.

## Introduction

Cystic adrenal lesions often present with nonspecific clinical and radiological features, leading to their under-recognition. Adrenal cysts are not so common. Studies have reported an incidence of adrenal cysts around 5.4% [1]. The count is even lower among the autopsy series, ranging from 0.064% to 0.18% [2]. These lesions are usually benign, non-functional, unilateral, and mostly occur in the third to sixth decade of life [3]. These cystic lesions can have significant clinical implications, particularly when they are associated with malignant neoplasms, but they resemble benign lesions. Although the literature on cystic adrenal lesions is limited, no cases of large adrenal cysts exceeding 20 cm have been reported from India so far.

## Case presentation

A 37-year-old female with no co-morbidities presented with complaints of gradually progressive abdominal distention and weight gain for a few years. She also had intermittent vague abdominal pain although her bowel and bladder habits were normal. There was no history of loss of appetite, fever, or vomiting. On examination, there was abdomen distension but there was no tenderness, guarding, or rigidity. She had pitting-type of pedal edema, and the distal pulsations over both lower limbs were normal.

**Figure 1:**
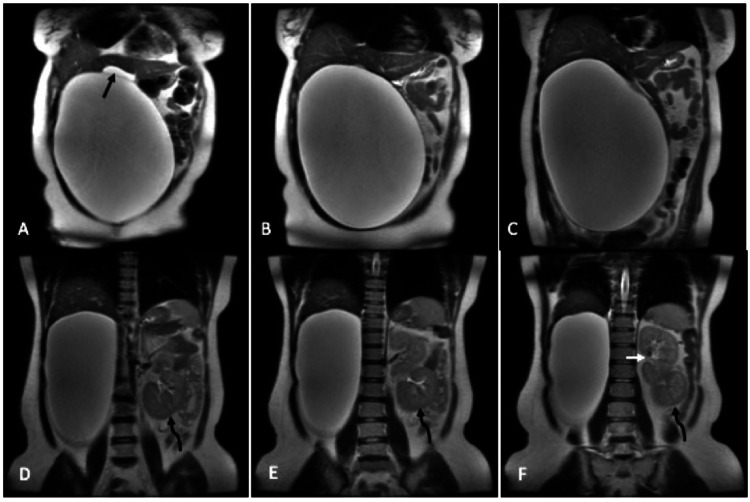
T2W coronal MRI images of the abdomen reveal a giant cyst extending from the right sub-hepatic region upto the iliac fossa and bulging across the midline with compression and displacement of the surrounding structures The black arrow in A shows the superiorly displaced and compressed gall bladder and curved black arrows in D, E, and F point toward the displaced right kidney into the left lumbar region below the left kidney (white arrow in F).

Routine laboratory tests showed no significant abnormality. Ultrasound of the abdomen showed a large cyst of size 20 × 25 cm extending from the right hypochondrium to the right iliac fossa. The right kidney could not be visualized in the right renal fossa, and there was a fused renal ectopia on the left. As the exact organ of origin of the cyst was unclear, we did a magnetic resonance imaging (MRI) of the abdomen for further evaluation. T2-weighted (T2W) MRI of the abdomen (Figure 1) revealed a large unilocular, thin-walled cyst, inseparable from the right adrenal and measuring 28.6 cm craniocaudally, 22.5 cm transversely, and 17.5 cm anteroposterior, extending from the right sub-hepatic region up to the right iliac fossa and bulging across the midline to the left.

**Figure 2:**
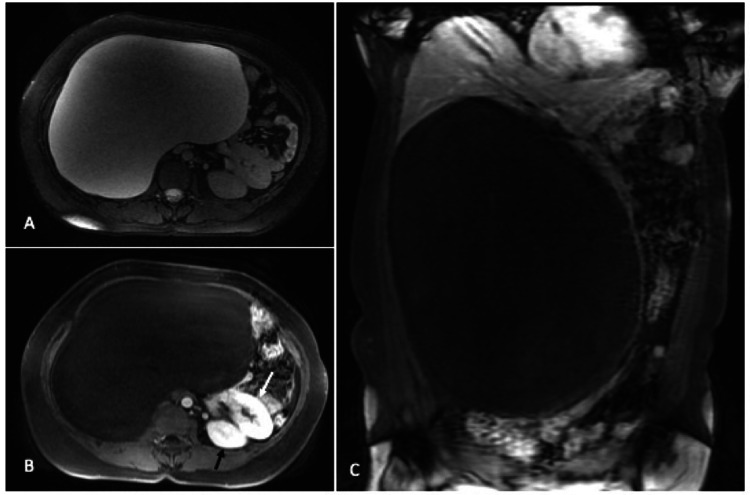
T2W axial (A) and fat-saturated T1W post-contrast axial (B) and coronal (C) MRI images reveal the giant right adrenal cyst with no evidence of septations or any enhancing solid component The displaced right kidney (white arrow in B) is seen anteroinferior to the left kidney (black arrow).

T2W (axial) and fat-saturated T1-weighted (T1W) post-contrast (axial and coronal) MRI images (Figure 2) revealed the giant right adrenal cyst had no septations or any enhancing solid component, although there was a severe displacement of the right-sided abdominal contents to the left. The right kidney was also located in the left lumbar region, below the left kidney. There was no evidence of ascites or lymphadenopathy. The rest of the abdominal structures were otherwise unremarkable. Based on these findings, it was evident that the giant retroperitoneal cyst was arising from the right adrenal.

**Figure 3:**
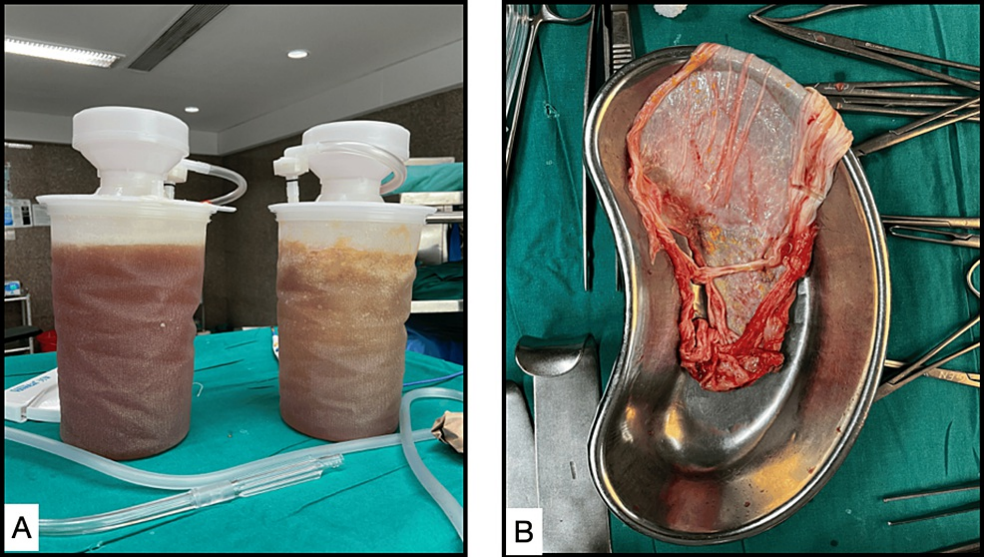
(A) Serosanguinous lymphatic fluid with minimal blood tinge aspirated from the cyst. (B) Excised cyst wall after fluid aspiration

The patient underwent exploratory laparotomy. Intraoperatively, the cyst was arising from the right adrenal and occupying the whole abdominal cavity. We aspirated around 3.5 liters of serosanguinous lymphatic cyst fluid with a minimal blood tinge (Figure 3).

Histopathological examination

**Figure 4:**
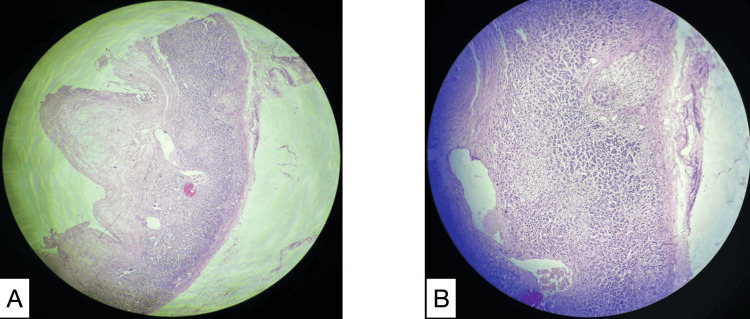
(A) 4x magnification of the excised cyst wall showing cells of adrenal origin. (B) 10x magnification of the excised cyst wall The cyst wall was lined by flattened endothelial cells with focal hemorrhages and the adrenal parenchyma was seen.

On gross examination, the cyst wall measured 20 × 12 cm with a smooth, white external surface and an inner surface with yellow granular areas. Microscopic examination showed the cyst wall lined by flattened endothelial cells with focal hemorrhage and adrenal gland parenchyma (Figure 4).

The postoperative period was uneventful, and the patient recovered well. There was a significant reduction in her weight and abdominal girth.

## Discussion

Cystic lesions of the adrenal gland are rare. They may be true cysts, infectious cysts, malignancies with cystic degeneration, or pseudocysts [4]. The earliest documented adrenal cyst was by Doran in 1670, attributed to Greiselius, a Viennese physician [5].

Due to the increased access to various imaging modalities, the incidence of adrenal pseudocysts has increased over the past few years. Only 7% of the reported pseudocysts are malignant or have malignant potential, and there is an increased risk with an increase in size over 6 cm [6,7]. Adrenal pseudocysts are supposed to be the result of hemorrhage into non-neoplastic adrenal tissue. Certain theories postulate that it is primarily of vascular origin and immunohistochemical studies have supported this theory. According to other theories, pseudocysts represent the end-stage of lymphangiomatous lesions that undergo hemorrhage or degenerative change and are replaced by fibrous tissue, whereas other etiologies postulated include changes in adrenal venous structures or blood vessel microvasculature [8,9].

Adrenal cysts can occur at any age, although they are more common in young adults and slightly more prevalent in females. Their size can vary significantly, ranging from one millimeter to over 4 cm, with very few exceeding 10 cm, but cysts are incidental findings when smaller than 10 cm and asymptomatic. However, symptomatic cases arise with giant adrenal cysts larger than 10 cm, as seen in this instance [10,11]. Adrenal cysts are typically solitary, uniloculated, or multiloculated and affect both the right and left adrenal glands equally [12,13]. While the clinical presentation of these benign cysts depends on their location and largest dimension, they are usually asymptomatic. However, in the case of larger cysts, symptoms such as dyspnea, abdominal pain, gastrointestinal disorders, and occasionally, a palpable mass may be present. Rarely, complications such as intracystic hemorrhage, infection, and hypertension may occur [12].

Histopathological types of adrenal cysts include epithelial cysts, parasitic cysts (specifically the echinococcal subtype), pseudocysts, and endothelial cysts [14]. Diagnosis of adrenal cysts begins with assessing the presence of a cyst lining. If absent, it favors a diagnosis of adrenal pseudocyst, characterized by fibrino-hemorrhagic fluid, and surrounded by thickened fibrous tissue. If a lining is present, further evaluation determines whether the cyst contains a monolayer of endothelial or mesothelial cells or flattened epithelial cells. Flat endothelial cells are associated with a lymphatic lining, sometimes with intraluminal papillary projections. Mesothelial cells are typically flat or cuboidal and usually lack atypia.

Among adrenal cystic pathologies, endothelial cysts and hematic pseudocysts are the most common. Hematic pseudocysts account for 40% of all cystic lesions in the adrenal gland and are lined by a thin capsule, isolating the region from the surrounding normal adrenal parenchyma [14]. Hematic pseudocysts are typically unilocular and contain reddish-colored fluid, often reaching large volumes. There have been reports of pseudocysts containing up to 1 liter of fluid [15].

Recently, a case of a retroperitoneal cystic lesion (8×9×9 cm) in the region of the tail of the pancreas was reported, which on CT scan had multiple diagnostic possibilities and a definitive pre-operative diagnosis regarding the origin of the cystic swelling could not be made [16]. So, the lesion was excised via an upper midline laparotomy and eventual histopathological diagnosis confirmed it to be an adrenal pseudocyst.

Another study reported a case of a 31×17×16 cm giant unilocular cystic lesion closely attached to the left adrenal gland [17]. Due to the huge volume and the risk of malignancy, they did an open excision and the final histopathology confirmed it as an angiomatous adrenal endothelial cyst. Also, a case of suprarenal cyst measuring 17×16×11 cm on abdominal CT scan was reported with anterior displacement of the pancreas and inferior displacement of the left kidney; it was thought to originate from the left adrenal gland [18]. They removed the cyst completely with total adrenalectomy, and histological examination revealed it to be a benign epithelial adrenal cyst.

Currently, there are no established protocols for the management of adrenal cystic lesions. In cases where malignancy is clinically suspected or excessive hormone secretion is seen, a multidisciplinary team is essential. Surgical intervention is indicated for cystic lesions larger than 5 cm. Symptomatic presentations, including endocrine abnormalities and suspected malignancy, also warrant surgical intervention. Asymptomatic lesions measuring <5 cm can be monitored through imaging, although no screening algorithms have been established. Laparoscopic adrenalectomy remains the preferred method for managing adrenal cystic tumors [15].

## Conclusions

Adrenal cysts are uncommon clinical conditions that often resemble more serious lesions like adrenal malignancies and pheochromocytoma. Various imaging modalities, like ultrasonography, CT scan, and MRI, and histopathological examination are crucial for subclassifying the cystic lesion and establishing an appropriate diagnosis, which is essential for determining the optimal treatment. While immunohistochemical workup can aid in confirming the diagnosis, the key factor in reaching a conclusive diagnosis lies in analyzing the morphological characteristics of the cystic lesion.
